# Genomic resources for Australian alfalfa (*Medicago sativa* L.) genomics: reformatted reference genome, annotated variants, gene presence-absence and diversity analysis from genome re-sequencing

**DOI:** 10.1186/s12870-025-07941-5

**Published:** 2025-12-16

**Authors:** M.M. Malmberg, D.D. Suraweera, R.C Baillie, K.F. Smith, N.O.I. Cogan

**Affiliations:** 1 Agriculture Victoria Research, AgriBio Centre for AgriBioscience, Bundoora, VIC Australia; 2 Agriculture Victoria Research, Hamilton Centre, Hamilton, VIC Australia; 3 School of Agriculture, Food and Ecosystem Sciences, The University of Melbourne, Victoria, Australia; 4 School of Applied Systems Biology, La Trobe University, Bundoora, VIC Australia

**Keywords:** Lucerne, WGS, Crop pan-genome, Polyploid, Allele frequencies

## Abstract

**Background:**

Alfalfa (*Medicago sativa* L.), a globally significant forage crop, has faced limited breeding progress in recent decades. Several challenges hinder genetic gain in alfalfa, including its status as an outbreeding tetraploid species with pronounced inbreeding depression, high parent numbers in synthetic crosses resulting in limited genetic differentiation between cultivars and a lack of genomic resources to advance genomic breeding techniques in the species.

**Results:**

We aim to address some of these limitations by generating genomic resources for alfalfa improvement, including reformatting an allele-aware reference genome to remove duplicate haplotypes while retaining presence absence variation, genome annotation identifying genes and functional elements, SNP discovery and SNP variant effect prediction. The predicted gene set was expanded by the inclusion of RNA sequencing from multiple tissue types and stress treatments. Genetic diversity of 316 samples from seven commercially available cultivars relevant to Australian grazing systems was examined, including a population level analysis of gene presence-absence variation. There is little genetic differentiation between cultivars, with higher diversity within than between cultivars. Several genes were found to display presence-absence at the population level.

**Conclusions:**

These findings provide insights for alfalfa breeding programs and underscore the need for continued efforts in developing genomic tools to unlock the crop’s full potential.

**Supplementary Information:**

The online version contains supplementary material available at 10.1186/s12870-025-07941-5.

## Background

Alfalfa (*Medicago sativa* L.) is an autotetraploid (2n = 4x = 32) obligate outbreeder with severe inbreeding depression and high levels of diversity, even within commercial cultivars. Current breeding approaches use high parent numbers to create new cultivars, known as broad-based synthetic breeding, in part to mitigate inbreeding effects [1]. This is consistent with high overall genetic diversity in alfalfa cultivars and the presence of higher variation within than between populations found by several studies [2–4]. Relatively low genetic differentiation is found between geographically distant populations [5] and is expected to be even lower between commercially available cultivars relevant to Australian grazing systems. Low genetic divergence between populations, with high expected genetic diversity within populations, requires quantification of existing agriculturally relevant germplasm to guide future strategies and accelerate breeding.

In order to facilitate genomic breeding in alfalfa, adequate genomic resources are required. A high-quality reference genome was recently released [6], as an allele-aware assembly, constructing four haplotypes for each chromosome. However, using an allele-aware haplotype assembly for genotype calling may result in random mapping between haplotypes, reduced overall genome coverage depth, and increased computational difficulty in associating multiple haplotypes with the same genomic region, an important concern for polyploid genotyping, which often relies on allele depth to call genotypes. The benefits of the haplotype aware assembly can be retained by selecting one representative haplotype for each chromosome as the core and incorporating additional diversity regions from remaining haplotypes as presence-absence variants (PAV), similar to a pan-genome format. In this way, additional genomic information absent in the core assembly can be incorporated, which is especially important for gene content which has been shown to vary significantly between individuals [7–10] and can therefore vary between the haplotypes of a single individual. The *Brassica oleracea* pan-genome incorporated an additional nine genomes and found that 18.7% of genes displayed presence-absence variation between lines [8]. In sorghum, incorporating 354 accessions resulted in 53% of genes displaying presence-absence variation [7].

Highly heterozygous autopolyploid species present challenges not only in producing accurate reference genomes, but also in routine genotyping. Numerous software programs exist for genotyping, but many do not provide adequate tools for polyploids. GATK does allow polyploid calling but requires significant computational resources and time, relying on older versions of the software with significantly longer processing times. Freebayes offers polyploid calling and is a commonly used tool within the alfalfa research community, but tends to preferentially call homozygous genotypes [11] and false-positive homoeologous SNPs [12]. Many other tools for genotyping polyploids exist but require extensive computational resources [13], specific population structures such as F1 populations [14] or were developed for array-based data [15–17] and therefore struggle with low read depths resulting in high variance of depth ratios [18]. The R package polyRAD [18] calculates continuous allele dosages by estimating genotype prior probabilities from population parameters, making it heavily dependent on selecting the correct prior.

Using an allele depth approach to generate allele frequencies has the advantages of buffering against misclassification error [19] and enables greater flexibility, simplicity and integration in terms of data handling [20]. Additionally, both individuals and population sequences, can be processed with the same software for some degree of consistency, which is particularly important for genotype calling as different software tools result in significantly different sets of called variants [12]. This approach is not without drawbacks as it requires a moderate level of computational skill, does not have the benefit of weighted genotype scoring, and some downstream software might not cater to genotype calls in the form of frequencies. Nonetheless, using allele frequencies reduces overall error if genotypes are not called or imputed accurately [21] and was determined to be the preferred approach for genotyping and genomic selection in polyploid blueberry [19].

The objective of this study was to (i) reformat an existing allele-aware reference genome assembly by collapsing multiple haplotypes and retaining variable regions in a pan-genome format, (ii) extend gene identification and annotation through the use of publicly available RNA sequencing data, (iii) examine diversity and population structure in a set of seven cultivars commercially available in Australia, including gene presence-absence.

## Methods

### Reformatted reference genome

#### PAV identification

The publicly available allele-aware reference assembly by Chen, et al. [6] was reformatted to reduce redundancy while retaining variable regions, similar to a pan-genome format. The longest allele assembly for each chromosome was retained as the core of the reformatted genome and all remaining sequences put through ScanPAV [22] to identify variant regions absent in the core chromosomes. Identified variant regions were retained if they were > 1,000 bp in length as per software recommendations. ScanPAV outputs were then further filtered by running a BLASTN v2.12.0 of all identified PAVs against the core chromosomes as a database, with a percentage identity cut off of 80% and e-value cut off of 1e -10. Sequences that were ≥ 80% similar and with a hit length ≥ 50% were removed. PAVs were also trimmed to remove large partial hit regions, where the hit length was ≥ 1,000 bp, and retained if the remaining trimmed sequence length was ≥ 1,000 bp (i.e., if trimming resulted in a fragment < 1,000 bp, it was removed). The reformatted genome was assessed for completeness and duplication compared to the original allele-aware assembly using BUSCO v5.1.2 [23] with the embryophyta_odb10 database in genome mode.

#### Annotation

The resulting reformatted genome was annotated using the GenSAS v6 pipeline [24], with the aim to expand the set of predicted genes using publicly available RNA sequencing data from a range of tissue types and stress treatments. Sequencing data from 37 publicly available RNA libraries [25, 26] were aligned to the reformatted genome in GenSAS using TopHat and used for gene prediction. Full length pacbio transcripts from four samples were also sourced [27] for PASA refinement v2.4.1. The source and stress treatment of all RNA sequences can be found in Supplementary Material 1.

Using the GenSAS pipeline, repeat regions were identified and masked using RepeatMasker v4.1.1 and RepeatModeler v2.0.1. Gene predictions were performed using Augustus v3.4.0 with Arabidopsis as the species, and BRAKER2 v2.1.5 using the 37 RNA sequences, selecting the final gene set using EvidenceModeler v1.1.1 with default parameters. PASA v2.4.1 refinement was performed using full length pacbio transcripts. The refined predicted gene set was annotated using BLASTP v2.12.0 with an e-value of 1e-10 and DIAMOND v2.0.11 against the SwissProt dataset.

Genome annotations generated for the reformatted genome using GenSAS were compared to the genome annotations generated by Chen, et al. [6]. The two sets of annotation were kept separate due to partial overlap between numerous genes in the two versions. The co-ordinates of the gene motifs identified in the Chen, et al. [6] allele-aware assembly were subset to those found within the final reformatted genome and position coordinates were updated to match the reformatted genome to enable cross-referencing.

### Genotyping

#### Plant material

A total of 316 individual plants were selected from seven cultivars relevant to Australian conditions: Silverosa GT7 (47 samples), Stamina Torrens GT8 (42), Titan 7 (44), Stamina GT6 (46), Sardi 7 Series 2 (47), Force 7 (45) and Force 7-2 (45). All seven cultivars were commercially sourced and are winter active varieties with good grazing tolerance.

#### Library preparation and variant discovery

Whole genome libraries were prepared by extracting genomic DNA from freeze dried leaf tissue using sbeadex (LGC Biosearch Technologies, UK) according to the manufacturer’s instructions. Whole genome DNA sequencing libraries were prepared using NEXTFLEX^®^ Rapid DNA-Seq Kit 2.0 (Revvity, USA) according to the manufacturer’s protocol. Samples were quantified for pooling using an Illumina MiSeq (Illumina, San Diego, CA) Nano 300 cycle run. Libraries were sequenced on an Illumina NovaSeq6000.

Reads were trimmed using quadtrim [28] and aligned to the reformatted reference genome described above, using BWA mem v0.7.17 [29] and SAMtools v1.9 [30]. Variant discovery was performed separately using both GATK v4.1.3 [31] and BCFtools v1.9 mpileup [30] with default parameters. After initial variant discovery, the VCF files generated from GATK and mpileup were separately filtered using VCFtools v0.1.16 [32] for biallelic SNPs only, removing indels, a minimum minor allele frequency (MAF) of 0.02, a minimum read depth of 5 and maximum missing data of 50%. SNPs which were common between the two sets, including both the reference and alternative alleles, were retained. Identified variants were annotated using SnpEff v5.0 [33].

The variant discovery and initial variant filtering steps were based on diploid calls. As alfalfa is an autotetraploid, reference allele frequencies were calculated in R [34], based on a VCF generated by the mpileup pipeline. Briefly, the allele depth (AD) field was extracted from the VCF and the number of reads for the reference allele was divided by the total number of reads at that position, resulting in a reference allele frequency ranging from 0 (no reference alleles) to 1 (all reads were the reference allele). The genotype calls were further filtered in R for a minimum read depth of 5, a minimum MAF of 0.05 and removing SNP loci with more than 25% missing data in samples with less than 50% overall missing data.

#### Gene presence-absence

To score gene presence-absence, each sample was individually processed using SGSGeneLossv0.1 [35], with lostCutoff = 0.1 and additionally a minimum of 50 bp coverage cutoff for presence-absence calling. Meaning that each sample must have a minimum 10% of total exon length covered and at least 50 bp coverage of exons to be considered present in that sample’s genome.

Gene presence-absence was examined on a population level by counting the number of individuals with a gene in each of the seven cultivars. A chi-squared test was performed on each gene across all seven populations, as well as on individual populations for each gene. Any gene with a p-value < 0.05 across all populations or a p-value < 0.01 for at least one of the populations was selected as having statistically significant variation. Finally, any genes which were completely absent in at least one population were incorporated.

The GO terms for genes displaying presence-absence were examined by running a BLAST of each predicted gene protein sequence against the UniRef90 database. Only the top hit was returned, requiring a minimum 0.01 e-value, and the UniProt ID mapping tool (https://www.uniprot.org/id-mapping) was used to retrieve GO terms. The full list of GO terms was condensed using REVIGO [36] for each of the three GO categories: Molecular Function, Cellular Components and Biological Process. The top 10 results from each category were plotted using https://www.bioinformatics.com.cn/.

#### Diversity analysis

To better understand population structure of Australian relevant germplasm, a diversity set representing global germplasm was sourced from Chen, et al. [4]. A total of 15 whole genome sequenced samples were selected to represent primarily cultivars from each major branch of the Chen, et al. [4] neighbour-joining (NJ) tree. This included four Chinese samples, four North American samples, five European samples, one South American sample and one Asian sample not from China. The details of the selected samples are provided in Supplementary Material 2. The downloaded genomes were processed using the same pipeline for genotype calling using mpileup as described above, calling the c. 1.13 million SNPs previously discovered and further filtering once combined with the 316 samples generated in this study.

The package StAMPP v1.5.1 [37] was used to calculate Nei’s D genetic distance. A Principal Co-ordinate Analysis (PCoA) plot and a NJ dendrogram were constructed from the Nei’s D genetic distance and plotted in R using ape v5.7-1 [38] and ggtree v3.8.2 [39]. The NJ tree had branch length set to “none” to only view the tree topology and distinguish points of branching, with the PCoA capturing genetic distances between individuals. StAMPP was also used to perform an AMOVA to compare within and between population variance, and to calculate Fixation Index (F_ST_).

## Results

### Reformatted reference genome

#### PAV identification

**Fig. 1:**
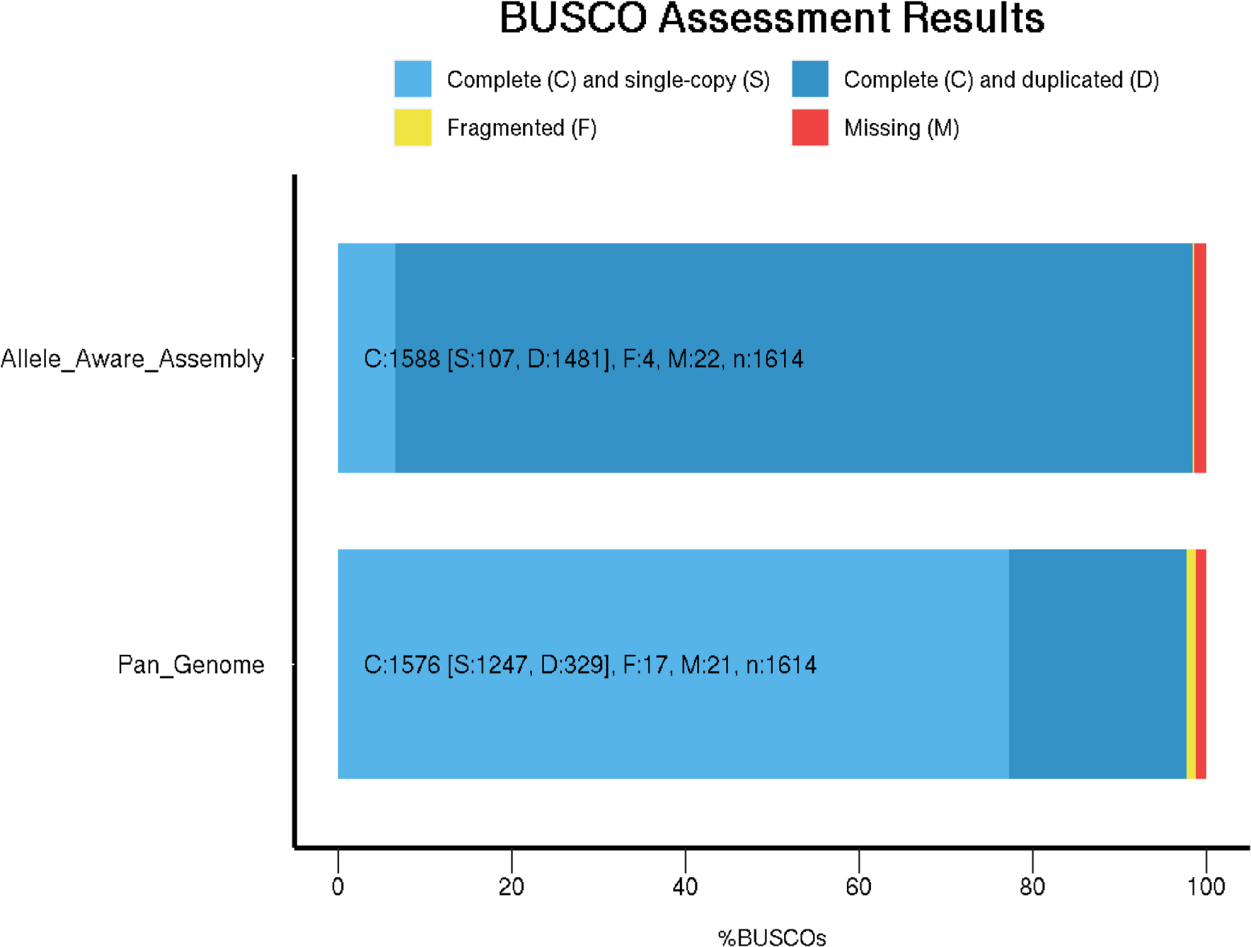
BUSCO notation (C: complete [S: single-sopy, D:duplicated], F:fragmented, M:missing, n:gene number) of the original allele-aware assembly and the reformatted genome

As there was little difference in alignment rate found between the four allele assemblies of each chromosome, the longest allele was selected as the core of the reformatted genome. Excluding contigs, the average chromosome length was 85.5 million bp, with chromosome 3 having the longest allele assembly at 100.4 million bp. Most chromosomes varied by between 2.5 and 8.9 million bp between their longest and shortest allele assemblies, with the exception of chromosome 6, which had a difference of 25 million bp due to one particularly short allele assembly for chr6.4 (64 million bp). From the contigs and haplotypes which were not selected, ScanPAV identified 168,307 initial PAVs, with 95,856 PAVs greater than 1,000 bp in length. After additional filtering and trimming based on BLAST results, a total of 94,003 PAVs were retained and added to the eight core chromosome sequences. The source regions of the identified PAVs were evenly spread across chromosome allele assemblies (Table 1). BUSCO analysis shows that reformatting the reference genome from the allele-aware assembly to a pan-genome format reduced duplicated gene content from 91.8% to 20.4%, with minimal fragmentation (0.2% to 1.1%) and gene loss (1.4% to 1.3%: Fig. 1).

**Table 1:** Length of the longest haplotype per chromosome and the number of presence-absence variant (PAV) regions incorporated into the reformatted genome

Source region	Longest Haplotype (bp)	No. PAVs
Chr1	88,815,615	10,217
Chr2	76,750,018	9,200
Chr3	100,414,524	11,445
Chr4	93,947,428	11,071
Chr5	84,165,483	10,337
Chr6	89,579,199	11,016
Chr7	94,657,719	11,825
Chr8	87,242,343	10,410
Contigs	NA	8,482
Total	715,572,329	94,003

#### Annotation

From the GenSAS pipeline, the predicted number of genes was 94,030, encompassing 93,525 proteins. Of these genes, 54,345 are found in the core chromosomes and 57,668 are found within PAV regions. The original Chen, et al. [6] allele-aware assembly has 164,632 predicted genes, of which a total of 46,325 genes were again identified in the reformatted genome, based on at least 50% overlap and having its full length captured in the reformatted genome. An additional 20,958 genes from the original assembly were not predicted as genes by the GenSAS pipeline but are located in regions which were retained as part of the reformatted genome. As a result, a total of 67,283 genes from the original assembly were lifted over and their position co-ordinates updated to match the reformatted genome structure and provided as a GFF (Supplementary Material 3). A large number of genes identified in the original assembly (97,349) were not incorporated, as the regions containing those genes were removed during the ScanPAV pipeline. Of the 94,030 genes predicted in the annotation of the reformatted genome, approximately half (46,325 or 49.3%) were common with the original assembly, and the other half (47,705 or 50.7%) represent newly predicted genes.

### Genotyping

Average genome coverage was 4.0x across all samples, ranging from 2.5x to 19.4x coverage. All samples were used for variant discovery, resulting in 90,641,278 variants from BCFtools mpileup and 92,162,031 variants from GATK. Initial filtering resulted in 1,699,140 SNPs from mpileup and 4,592,856 from GATK, of which 1,135,370 were common between both, including the same REF and ALT alleles. For downstream analysis, the genotype calls were first converted into continuous reference allele frequencies and further filtered in R as described above, resulting in 245,708 SNPs across all 316 samples and 11% missing across the genotype matrix. The minimum, 25th percentile, median, 75th percentile and maximum missing data were 0.03%, 5.69%, 10.35%, 16.37% and 28.97% for individual samples, and 0%, 3.48%, 10.76%, 18.35% and 25% for SNP markers.

**Fig. 2:**
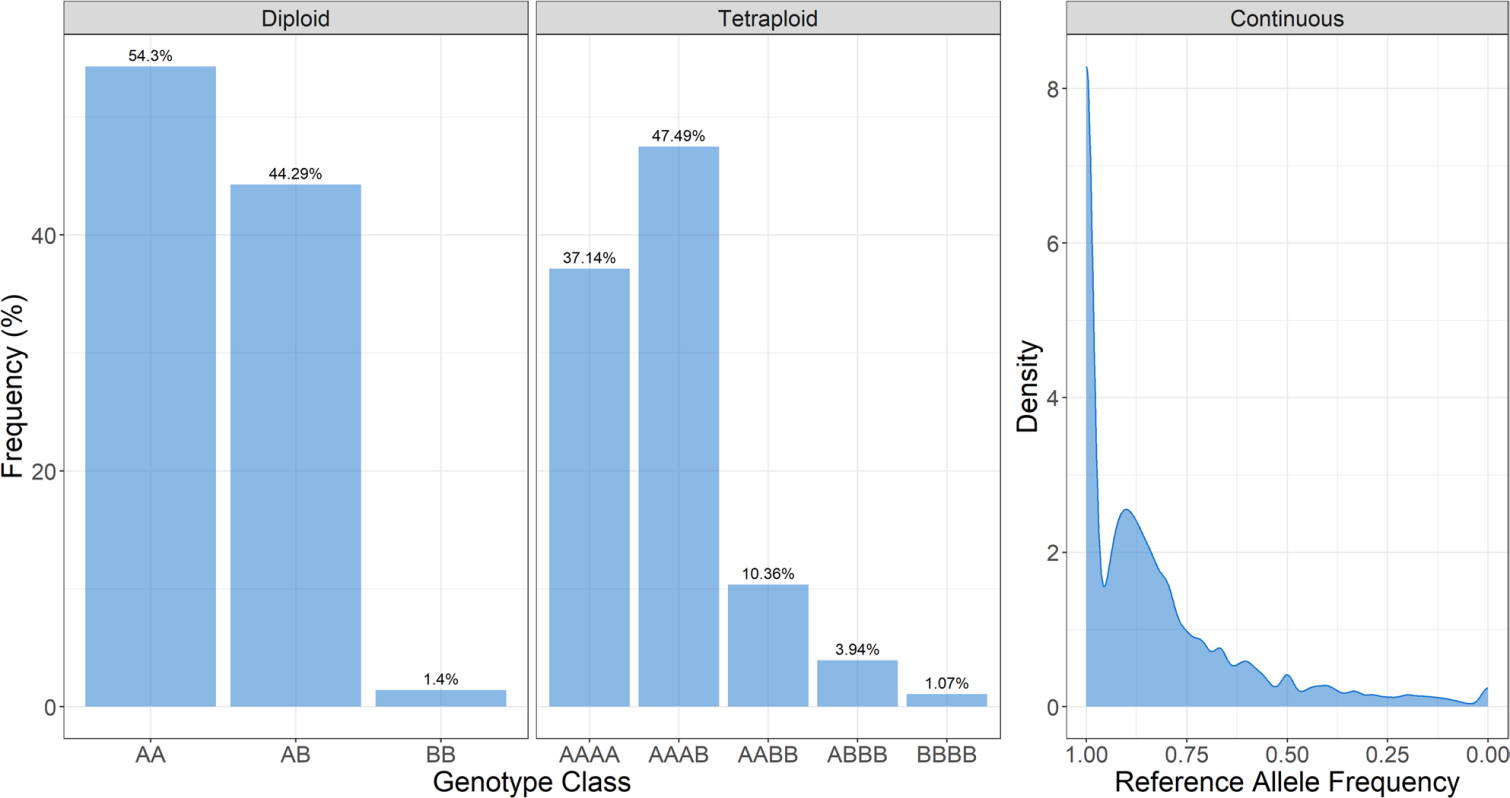
Frequency distribution plots of genotypic classes called using (A) diploid calls from mpileup, (B) binned tetraploid classes based on allelic depths and (C) non-binned allele frequencies based on allelic depths

The effect of genotyping approach on called genotypes and rates of heterozygosity was examined (Fig. 2). Using diploid calls from BCFtools mpileup resulted in 44.23% heterozygous calls, while binning allele frequencies into the five expected tetraploid genotypic classes based on allelic depths resulted in 61.79% heterozygous calls. Continuous allele frequencies are also calculated from allelic depth information but are not binned into discrete classes. Assuming, based on empirical evaluation, that the classification cutoffs for a heterozygous call is somewhere between 0.01 and 0.05 and 0.95–0.99, between 61.43% and 64.33% of continuous genotypes are heterozygous.

All 1.13 M SNPs identified by both GATK and BCFtools mpileup were annotated using SnpEff, with full results available in Supplementary Material 4. The majority of SNPs were modifiers (89.15%), with only 0.27% predicted to have a high impact. When PAV regions and core chromosomes are examined separately, there is a marginally higher percentage of high impact SNPs located in PAV regions (0.55%) compared to chromosomes (0.23%), noting that the PAV regions contain c. 11% of total SNPs identified (131,140 out of a total of 1,135,368). Across the whole reformatted genome, just under half of all SNPs are predicted to be silent (46.04%), over half are missense (52.08%) and a small proportion are nonsense (1.88%). In line with predicted SNP impact, PAV regions harbour a higher percentage of both nonsense (3.86%) and missense (57.35%) effects compared to chromosomes (1.64% and 51.45% respectively). Despite having higher impact SNPs, PAV regions primarily see an increase in intergenic SNPs, as well as a marginal increase in exon SNPs (Table 2), and a reduction in upstream and downstream SNP effects. Genes located in PAV regions are more likely to contain variants within the gene compared to the chromosomes.

**Table 2:** Summary of SnpEff annotations of reformatted genome

	Pan-Genome		Chromosomes		PAV Regions	
	Count	%	Count	%	Count	%
Downstream	383,249	19.91	346,639	20.10	36,610	18.34
Exon	204,115	10.61	182,115	10.56	22,000	11.02
Intergenic	846,153	43.97	747,798	43.36	98,355	49.27
Intron	120,622	6.27	109,738	6.36	10,884	5.45
Splice site acceptor	438	0.02	382	0.02	56	0.03
Splice site donor	345	0.02	297	0.02	48	0.02
Splice site region	3,578	0.19	3,197	0.19	381	0.19
Upstream	360,787	18.75	329,493	19.10	31,294	15.68
UTR 3 prime	2,781	0.15	2,774	0.16	7	0.00
UTR 5 prime	2,414	0.13	2,411	0.14	3	0.00
**Total**	1,924,482	100	1,724,844	100	199,638	100

#### Gene presence-absence

A gene presence-absence matrix is provided in Supplementary Material 5. Just over half (51.7%: 48,627) of genes were detected in all samples and 75.1% of genes (70,630) are present in 299 of 316 (94.6%) samples. Core genes present in all samples had a larger total exon length and gene size than dispensable genes on average. Gene content varied between samples, ranging from 83,192 to 90,466 genes present, but was correlated with genome sequencing coverage (0.77). On average, samples had sequencing coverage of 95.9% of genes located on the core chromosomes and 85.8% of genes located in the PAV regions. A total of 619 genes were not detected in any samples, primarily due to filtering cut offs removing 559 genes with small exon regions (< 50 bp). A total of 60 genes were not detected in any samples, even though they have exon regions > 50 bp in length, including large genes (> 3 kb). Twelve genes had total gene size > 3 kb but exon size < 50 bp, suggesting that these genes may contain large repetitive elements. Most undetected genes are located in PAV regions, with only 18 located in the core chromosomes.

**Fig. 3:**
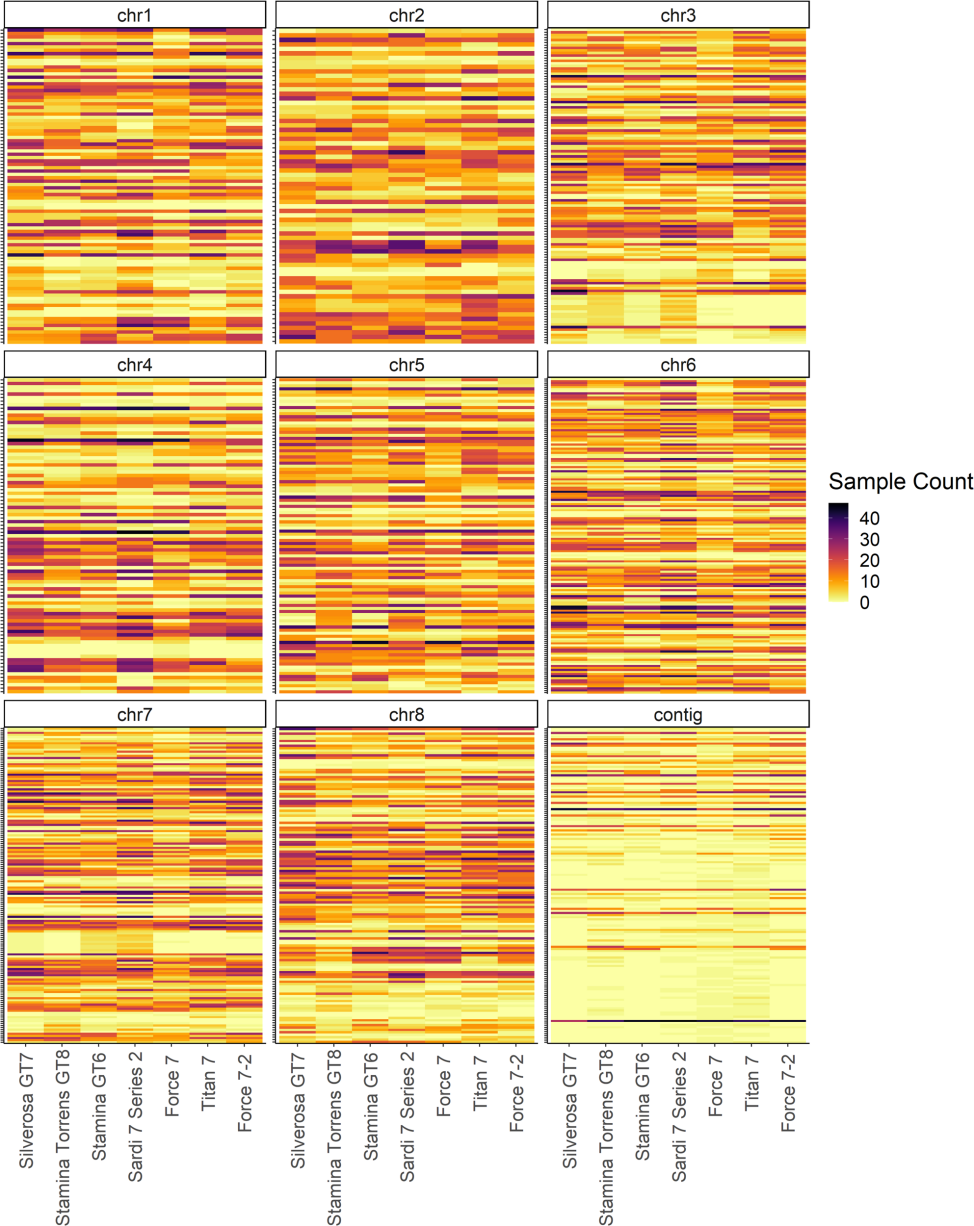
Gene presence-absence variability across seven cultivars of alfalfa. The y-axis represents each gene detected with presence-absence variation across populations, with gene names removed to improve clarity of figure

**Fig. 4:**
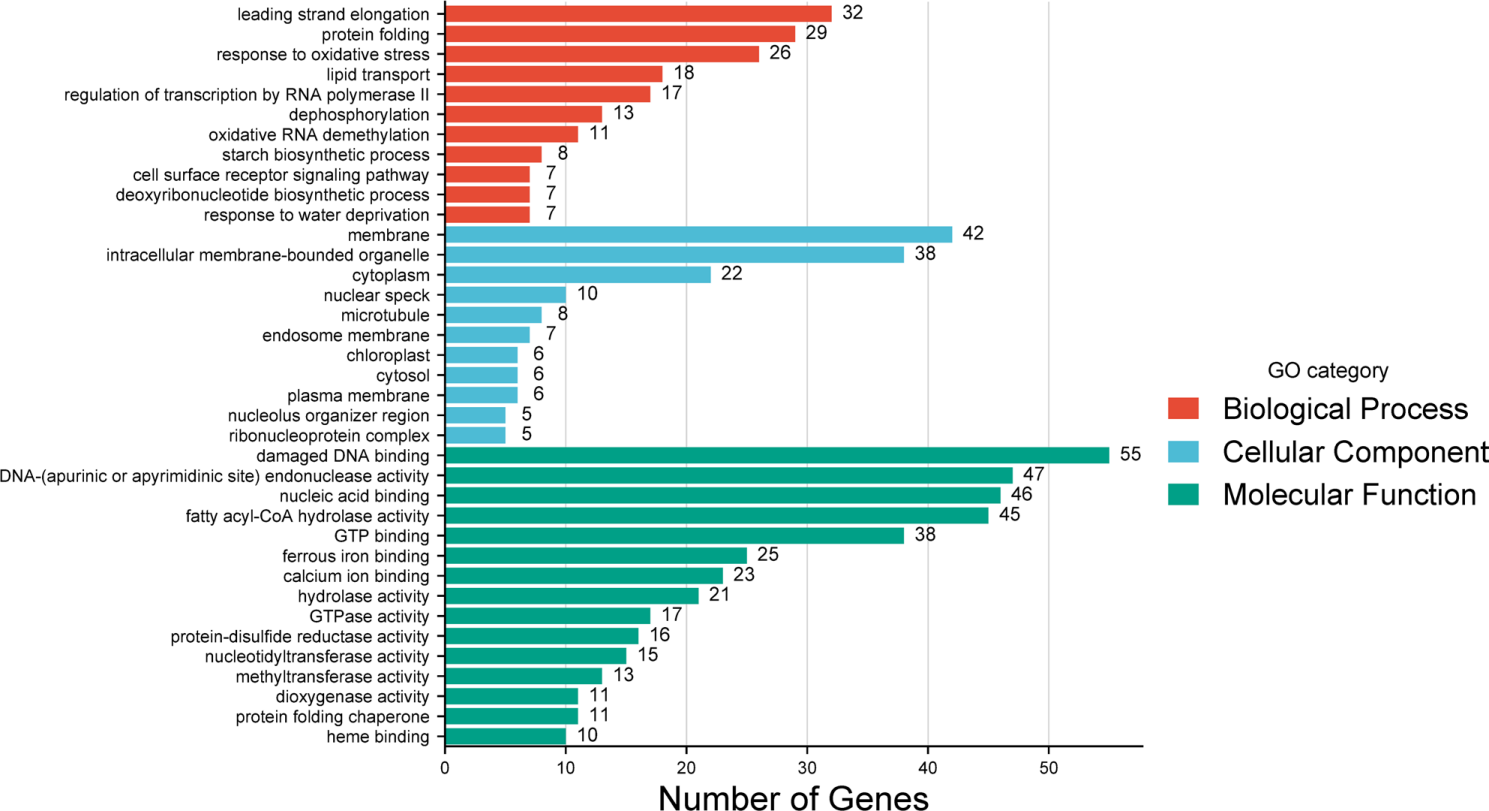
Top 10 most common gene ontology terms within each GO category found in genes displaying presence-absence variation across seven cultivars of alfalfa

At the population level, the majority of genes were detected in all seven cultivars. A total of 1,062 genes showed significant presence-absence variability between populations (Fig. 3), of which 453 were completely absent in at least one cultivar, 497 displayed significant variation across cultivars (p-value < 0.05) and 112 displayed highly significant variation from expectation in at least one cultivar (p-value 0.01). Of the 1,062 genes showing significant population presence-absence variation, 632 had a hit against the Uniref90 database, of which 315 genes had at least one associated GO term. GO terms were summarised and displayed in Fig. 4. The most common terms associated with Biological Process, Cellular Component and Molecular Function were leading strand elongation [GO:0006272], membrane [GO:0016020] and damaged DNA binding [GO:0003684], respectively. Some of the Biological Process terms of interest in gene PAVs include methylation and demethylation, regulation of flower development, reproductive structure development, embryo development ending in seed dormancy, defence response, as well as environmental responses including responses to water deprivation, heat, cold, biotic stimulus, other organisms, and symbiotic fungus. The full set of GO terms found for gene PAVs is provided in Supplementary Material 6.

#### Diversity analysis

**Fig. 5:**
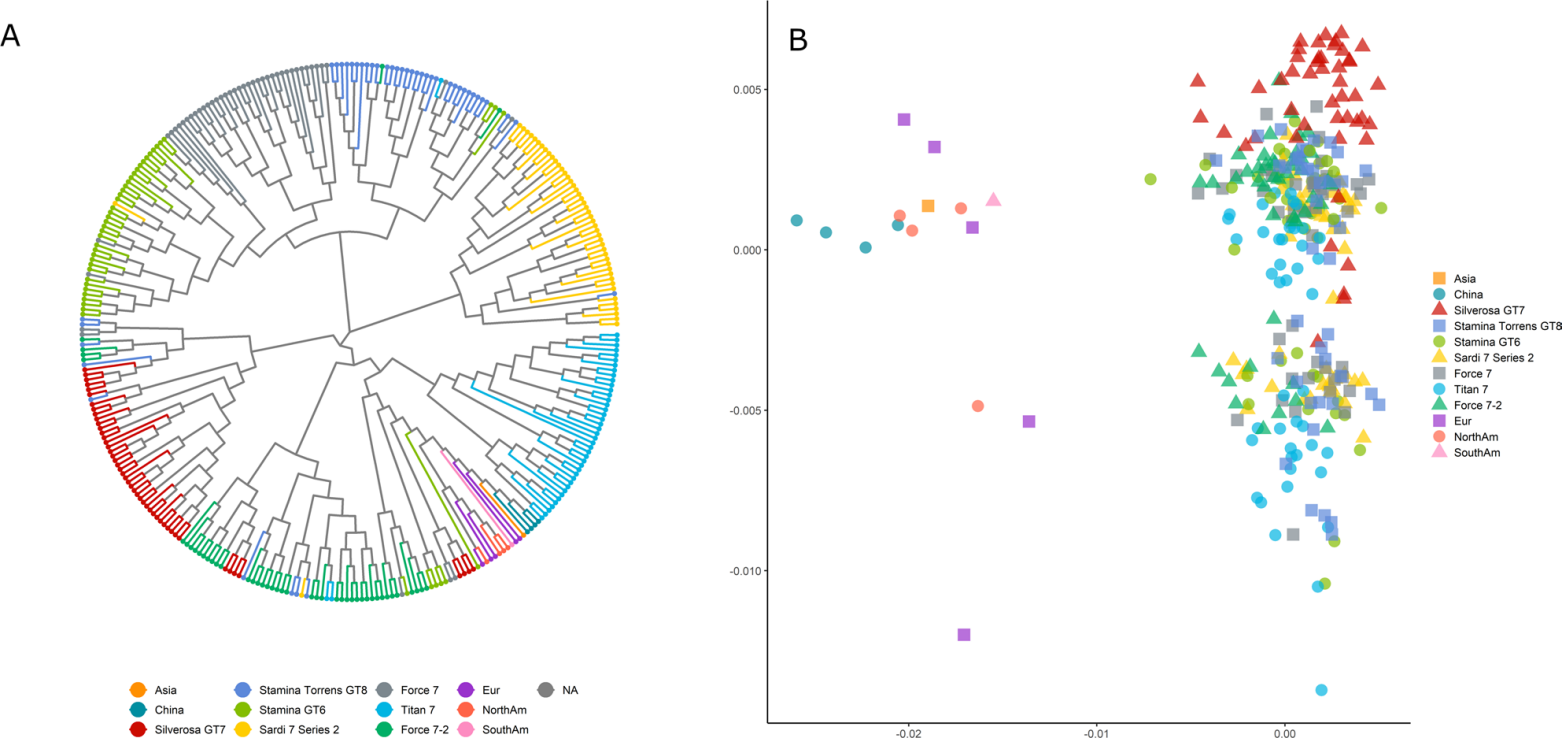
Neighbour joining dendrogram and PCoA plot of seven Australia-relevant cultivars and a diversity set of 15 international varieties of alfalfa

A NJ tree and a PCoA plot were constructed based on Nei’s genetic distance (Fig. 5). Although cultivars tend to cluster, some individuals did not cluster with their source population, and all individuals root to the same central point. The PCoA plot shows that all Australian cultivars cluster close together, with significant overlap. The international diversity germplasm sourced from Chen, et al. [4] sits distinctly apart from the Australian commercial cultivars as expected, both in the NJ tree and the PCoA plot.

The lack of distinction between Australian cultivars is further supported by the F_ST_ results. Australian cultivars had a mean pairwise F_ST_ of 0.0041 (*P* = 0.000) between cultivars (Table 3), indication almost complete panmixis. The lowest F_ST_ was between Stamina GT6 and Force 7 at 0.0028. The highest F_ST_, at 0.0051, was between Silverosa GT7 and Sardi 7 Series 2, as well as Titan 7, indicating some divergence between these populations, but the value is so low as to be near panmixis.

**Table 3:** F_ST_ values between the seven cultivars examined in this study

	Silverosa GT7	Stamina Torrens GT8	Stamina GT6	Sardi 7 Series 2	Force 7	Titan 7	Force 7 − 2	North America	China	Europe	Asia	South America
Silverosa GT7	NA	NA	NA	NA	NA	NA	NA	NA	NA	NA	NA	NA
Stamina Torrens GT8	0.0047	NA	NA	NA	NA	NA	NA	NA	NA	NA	NA	NA
Stamina GT6	0.0042	0.0038	NA	NA	NA	NA	NA	NA	NA	NA	NA	NA
Sardi 7 Series 2	0.0051	0.0045	0.0041	NA	NA	NA	NA	NA	NA	NA	NA	NA
Force 7	0.0043	0.0037	0.0028	0.0041	NA	NA	NA	NA	NA	NA	NA	NA
Titan 7	0.0051	0.0047	0.0042	0.0050	0.0042	NA	NA	NA	NA	NA	NA	NA
Force 7 − 2	0.0041	0.0038	0.0032	0.0044	0.0032	0.0038	NA	NA	NA	NA	NA	NA
North America	0.0220	0.0233	0.0192	0.0221	0.0192	0.0220	0.0220	NA	NA	NA	NA	NA
China	0.0306	0.0320	0.0271	0.0296	0.0271	0.0306	0.0306	0.0425	NA	NA	NA	NA
Europe	0.0190	0.0202	0.0160	0.0185	0.0161	0.0186	0.0181	0.0294	0.0351	NA	NA	NA
Asia	0.0411	0.0473	0.0332	0.0404	0.0329	0.0444	0.0472	0.0941	0.0887	0.0725	NA	NA
South America	0.0430	0.0487	0.0350	0.0418	0.0352	0.0451	0.0477	0.0936	0.0979	0.0738	0.2116	NA

In contrast, when including the international diversity set, the highest F_ST_ was between South America and Asia. However, both of these population are represented by a single sample and are not an ideal comparison. Excluding these samples, the highest F_ST_ is observed between North America and China (0.0425), which is higher than the value observed in Chen, et al. [4] and is likely explained by our use of a small subset of the samples from the original study. All of the Australian cultivars are most similar to the European set of samples out of the international diversity set.

The AMOVA revealed that 2.4% (*P* = 0.000) of variation in the alfalfa samples is due to variation between populations, while 97.6% is due to variation within populations. If only Australian cultivars are examined, 1.7% (*P* = 0.000) of variation is due to variation between cultivars, while 98.3% is due to variation within cultivars. There is greater diversity within than between cultivars.

## Discussion

Alfalfa is an economically important crop globally and genomic resources are required for the improvement of this species. In this manuscript, significant contributions have been made to the alfalfa genomic resources available for Australian alfalfa breeding, by reformatting an allele-aware chromosome assembly into a pan-genome format, additional gene annotation, generation of a large collection of re-sequenced whole genomes for the species, generation of an annotated SNP list, and analysis of genetic diversity in commercial germplasm including gene presence-absence variation.

### Reformatted genome

The reformatted genome developed in this manuscript is similar to a single sample pan-genome but differs from a traditional pan-genome, which incorporates variant regions from multiple individuals. While it incorporates the diversity present between the four alleles in the autotetraploid alfalfa genome of a single individual, it cannot fully distinguish between core and dispensable genes. Nonetheless, genes incorporated in the PAV regions are less likely to be involved in essential plant functions as compared to genes included in the core chromosomes. If a gene is truly non-dispensable, it is likely to be located on all four alleles and would be captured in the core of the reformatted genome or risk being lost during segregation. There is some evidence that genes located in PAV regions of this reformatted genome are more dispensable than those located in the core chromosomes, with only 4.1% of genes in the core chromosomes absent in our samples on average, while the same figure is 14.2% in PAV regions. To fully expand and characterise core and dispensable genes in alfalfa, PAV identification from multiple individuals needs to be performed, as has been done in several other plant species [7–9, 40] and coupled with transcriptome analysis for high confidence gene prediction.

A recent study by Kaur, et al. [41] generated a graph-based pan-genome using 3 chromosome scale assemblies of alfalfa and compared SNPs and structural variants between them. Predicted SNP effects from Kaur, et al. [41] are similar to those found in this study, except for a higher percentage of exon versus intergenic SNPs and is likely due to the different nature of the genomes and SNP sets. This study used a reformatted genome in the format of a within individual pan-genome but called SNPs across a large diversity set while Kaur, et al. [41] makes a direct comparison between 3 chromosome scale assemblies. Potentially of greater interest is the impact of structural variants, with Kaur, et al. [41] finding that structural variants are predicted to have a higher impact on genes than SNPs. This is in line with our observation that there is gene presence-absence between individuals and populations of alfalfa. While current downstream software is not yet able to easily parse graph-based genotypes, their analysis highlights the extent of potential differentiation between individuals of the same species, even when sourced from the same broad geographical region, and therefore the value of generating pan-genome format resources.

This study identified additional genes which were not present in the original assembly by using a set of publicly available RNA sequences sourced from multiple tissue types (root, stem, and leaf) and stress treatments (control, salt stress and drought stress). The original Chen, et al. [6] assembly used a range of plant gene databases as well as RNA sequencing from both root and shoots of the plant used to generate the assembly. While the inclusion of RNA sequences from stress treatments explains some of the additional genes identified, the choice of gene prediction software is also likely to have had a significant effect on the gene sets identified. Further refinement of predicted gene would be of value in alfalfa. Although a large number of genes from the original assembly were not incorporated into the reformatted genome, they are expected to represent duplicated genes or alleles of the same gene, with unique genes retained. As such, the removed genes primarily do not represent gene loss so much as a reduction in duplication, as could be seen in the BUSCO analysis. Nonetheless, some gene fragmentation was detected, indicating that some genes were partially removed in the PAV identification process. If gene variants or families exist where a portion of the gene is conserved and the other is variable, only the variable portion of the gene would be incorporated in the reformatted genome, resulting in a truncated gene sequence. The same is likely to occur in any PAV approach where genes are not first identified and deliberately kept intact. There was also a cohort of genes identified in the original allele-aware assembly which were not identified in the GenSAS pipeline, but which could be mapped to a position in the reformatted genome, highlighting the imperfect nature of current gene prediction tools.

### Genotyping approach

Software catering to polyploid genotyping is understandably lacking compared to diploids due to the additional complexity of detection of high number of potential genotype classes, such as the five genotype classes possible in an auto-tetraploid. While using diploid calls on polyploid individuals can yield good results for genomic selection and enables the use of lower overall sequencing depth to determine heterozygosity [21], the number of heterozygous genotype calls varies significantly between diploid and polyploid genotype calls, as seen both in this study and in blueberry [19]. We found that the majority of genotype calls were the reference allele, with a few alleles of intermediate frequency, in line with Julier, et al. [42]. Using relative read depth of the reference and alternative alleles to call allele frequencies has the advantage of buffering any error which is incorporated into either observed or imputed genotypes by reducing misclassification, increasing the accuracy of downstream analysis such as genomic selection [19, 21]. Another study in *Urochloa* interspecific tetraploid hybrids found that although tetraploid calls and diploid calls varied in predictive ability depending on the trait, tetraploid calls were better at selecting the top 10% of high performers as determined by phenotypic selection [43]. The variability in prediction accuracy observed in the Matias, et al. [43] study might be explained by the use of discrete tetraploid genotype classes and a kinship matrix to perform genomic prediction, rather than continuous allele frequencies and Bayesian genomic prediction [19].

In this study, we saw that PAV regions are more likely to contain SNPs within genes compared to the core chromosomes, as well as a higher proportion of both nonsense and missense mutations. Retroelements have been found to play a significant role in the variation in the dispensable genomes of different individuals of the same species, which includes insertion of complete or truncated genes [44]. Conversely, a higher proportion of synonymous SNPs were observed in genes on the core chromosomes. In general, the proportions of predicted SNP effect types (intergenic, synonymous versus non-synonymous) match the findings of the sorghum pan-genome [7] for both core and variable genes, assuming that genes located on the PAV contigs in this reformatted genome can be thought of as dispensable or variable genes and those located on the core chromosomes as core genes.

#### Gene presence-absence

Although SNPs are one of the most common variants used for genotyping, other variant types such as insertions, deletions, copy number variation and PAVs can be highly informative. A study in Arabidopsis showed that using either SNPs or gene PAV resulted in similar population structure identification, indicating a strong correlation between gene PAV patterns and individual variation [45]. Despite this relationship, many genes which are either genomic PAVs or expression PAVs, are not in strong linkage disequilibrium with neighbouring sequence variants [46]. It is known that the presence or absence of genes can determine agronomically relevant traits such as disease resistance [47], fruit morphology [48] and growth period structures [49]. Not only is the presence or absence of a gene itself relevant, but PAV regions harbour more variants in general, making them of high interest to genomic breeding.

Just over half of all genes were found in all samples examined in this study, with c. 75% of genes present in the majority of samples (94.6%). Core genes present in all samples also had a higher exon number and larger gene size than dispensable genes on average, in line with findings in other plant species [7, 8, 50, 51]. While some genes were absent in all samples, the majority of these genes had small exon regions (< 50 bp) and may represent fragmented genes, where the whole gene was not incorporated into the reformatted genome assembly. Although total gene content varied between individuals, this was highly negatively correlated with sequencing coverage. While low sequencing coverage has been found to be acceptable for several quantitative genetics approaches, such as genomic prediction, genome wide association studies, population differentiation and structure assessment [21], this analysis would benefit from deeper sequencing coverage or targeted methods for determining gene presence-absence to increase confidence. For this reason, gene PAV was examined on a population level, with more accurate methods needed to detect gene PAVs reliably and incorporate this information into downstream genomic analyses. A relatively small number of genes were found to display statistically significant variation (1.1%), as compared to 18.7% and 53% in *B. oleracea* [8] and sorghum [7] respectively, but this number is expected to be higher if examined in individuals rather than populations, especially considering the high genetic diversity of alfalfa. The primary factors affecting the degree of gene PAVs is expected to be overall species diversity and sample number, with 10 samples used in the *B. oleracea* pan-genome and 354 samples used in sorghum.

The population-based gene PAVs were examined in terms of GO annotation, finding several terms for environmental responses including responses to water deprivation, heat, cold, biotic stimulus, other organisms, and symbiotic fungus. Dispensable or variable genes are often involved in agronomic traits, particularly plant defence response [7, 8, 52–54]. Epigenetic effects may also be differentially regulated through gene PAVs, with terms including methylation and demethylation detected. Several reproductive GO terms were also detected including regulation of flower development, reproductive structure development and embryo development ending in seed dormancy. Although flower development genes are highly conserved across angiosperms [55], there is significant diversity in traits related to flower development, such as flowering time, which is thought to be due to complex downstream or independent genetic pathways [56, 57] which are more likely to be influenced by a cohort of variable genes. The wide geographical spread of alfalfa may account for the diversity and variability of reproductive and flowering related genes [4].

#### Diversity analysis

Numerous studies have already confirmed high genetic diversity of alfalfa, with significantly higher within than between population diversity [2, 4, 5, 42, 58–60], and is confirmed by the results in this study. Population differentiation is almost non-existent between the seven Australia relevant cultivars examined, and several breeding populations have been found not to have any population structure [61, 62]. In Australia, alfalfa was first brought over by settlers, likely from France, with seed produced up until the 1970 derived from this germplasm [63]. In this study, all of the Australian cultivars are most similar to European germplasm, in line with this breeding history. Even between highly geographically isolated populations only modest population structure exists such as between Egyptian, Italian and East European/Canadian/Meligo (EECM) populations [5]. Chinese varieties tend to differentiate most significantly from other germplasm [4, 59, 60].

## Conclusion

Substantial resources for the enhancement of genomics research and application in alfalfa have been made in this study. A pan-genome format reference has been developed from a single individual allele-aware chromosome level assembly to enable allele frequency based genotyping. Genetic variants in the form of SNPs have been detected and annotated, along with initial gene PAV identification and GO analysis. Diversity within commercial germplasm relevant to the Australian alfalfa industry has been examined and found to be significantly higher within than between populations. Substantial gene PAV has been identified across commercially available cultivars of alfalfa.

## Supplementary Information

Supplementary Material 1.

Supplementary Material 2.

Supplementary Material 3.

Supplementary Material 4.

## Data Availability

The pan-genome, associated annotation files, predicted SNP effects and gene presence-absence matrix can be found on figshare (10.6084/m9.figshare.28340273). The whole genome sequences can be found at NCBI under BioProject PRJNA1219757.
