# Supplementary material for: Genomic resources for Australian alfalfa (Medicago sativa L.) genomics: reformatted reference genome, annotated variants, gene presence-absence and diversity analysis from genome re-sequencing
Source: BMC Plant Biol. 2025 Dec 16;26:102. doi: 10.1186/s12870-025-07941-5 (PMC12822192; doi:10.1186/s12870-025-07941-5)
Supplement: Supplementary file 1 — Supplementary Material 1. [file 12870_2025_7941_MOESM1_ESM.docx]

Find additional supplemental materials on Figshare.

Private link – for reviewer access prior to publication

<https://figshare.com/s/833510f9aab9582e2548>
